# Evaluation of common carotid artery wall stiffness by shear wave elastography in smokers and non-smokers

**DOI:** 10.18332/tid/185300

**Published:** 2024-03-08

**Authors:** Kamber Goksu, Ahmet Vural, Ahmet N. Kahraman, Isil K. Aslan

**Affiliations:** 1 Department of Radiology, Fatih Sultan Mehmet Training and Research Hospital, University of Health Sciences, Istanbul, Türkiye; 2 Department of Neurology, Fatih Sultan Mehmet Training and Research Hospital, University of Health Sciences, Istanbul, Türkiye

**Keywords:** arterial stiffness, SWE, smoking, elasticity

## Abstract

**INTRODUCTION:**

Smoking is one of the most important preventable causes of cardiovascular diseases. Vascular disease caused by smoking is associated with vascular endothelial damage, platelet aggregation, and adhesion. In our study, we examined the effect of chronic smoking on vessel wall stiffness in smokers and control group by measuring carotid artery wall stiffness by shear wave ultrasonography.

**METHODS:**

Sixty-two smokers of similar ages and genders, and 67 people who never smoked in the last ten years were included as the control group in this cross-sectional study. Arterial wall stiffness over the common carotid arteries of all participants was measured by shear wave elastography (SWE). In addition, each patient's blood pressure, fasting blood glucose, body mass index (BMI), HDL and LDL cholesterol measurements were recorded.

**RESULTS:**

Arterial wall stiffness values in smokers were found to be statistically significantly higher than in non-smokers. The mean of SWE measurements of the smokers was 47.3 ± 6.2 kPa, and that of the control group was 42.9 ± 4 kPa. The mean values of HDL and LDL of the smokers were 46.9 ± 5.6 mg/dL and 147.3 ± 9.3 mg/dL, respectively, and those of the control group were 50.3 ± 5.1 mg/dL and 136.9 ± 5.9 mg/dL. The LDL cholesterol values were statistically significantly higher in smokers compared to the control group, and HDL cholesterol values were statistically significantly lower in smokers.

**CONCLUSIONS:**

In our study, the arterial wall stiffness values measured by the SWE technique were higher in smokers than non-smokers.

## INTRODUCTION

Despite the measures taken, morbidity and mortality due to smoking are increasing worldwide. The harmful effects of smoking depend on factors such as the years of smoking, how many cigarettes are smoked in total and per day, the content and filter structure of the cigarette, and the amount of smoke inhaled^1-3^. Smoking is one of the leading preventable risk factors for cardiovascular diseases^4^. Eleven percent of deaths due to cardiovascular diseases globally are related to smoking^5^. There are many diseases known to be caused by smoking: cancer, ischemic heart disease, stroke, and chronic obstructive pulmonary disease, are the most common^6^.

As a risk factor for cardiovascular diseases, smoking causes acute and chronic myocardial changes. In an acute state, smoking causes myocardial ischemia by increasing oxygen demand or decreasing oxygen delivery. These changes are thought to occur due to coronary artery spasm and/or platelet aggregation and adhesion, and changes in basal nitric oxide (NO) or endothelial nitric oxide synthase (eNOS) protein production. Chronically, smoking causes coronary atherosclerosis, possibly with recurrent endothelial damage. In addition, by stimulating smooth muscle proliferation, it increases platelet aggregation and adhesion, increases LDL cholesterol, and decreases HDL cholesterol^7-12^.

Mahmud and Feely^13^ demonstrated that smoking just one cigarette can cause an abrupt rise in arterial stiffness. Vlachopoulos et al.^14^ showed that smoking and caffeine consumption both cause a significant increase in arterial wall stiffness in a similar study. Increased carotid arterial wall stiffness has also been reported in non-smokers following exposure to environmental tobacco smoke as passive smokers^15,16^.

The arterial stiffness index is emerging as an important cardiovascular risk factor indicator due to new non-invasive technologies enabling measurement in large-scale clinical trials. In the past years, to measure arterial stiffness, the Pulse Wave Velocity (PWV) test, which aimed to determine the velocity at which the blood pressure pulse propagates through the circulatory system, was used as well as the systemic augmentation index (AIx) generated from the pulse wave obtained by applanation tonometry from peripheral arteries. The hardness index was determined by evaluating the changes in vessel diameter and area, and blood pressure measurement, together^17-20^. It has been reported that, with newly developing technologies, measurements made with shear wave ultrasound elastography (SWE) are quite successful in measuring *in vivo* tissue stiffness values^21^. There are no studies that compare SWE with traditional methods of measuring arterial wall stiffness. Comparative studies need to be conducted to demonstrate the reliability of SWE in measuring vessel wall stiffness.

In our study, we aimed to measure and compare the carotid artery wall stiffness of chronic smokers and control group non-smokers, to investigate the effect of smoking on arterial wall stiffness. We used the SWE method for arterial wall stiffness measurement.

## METHODS

### Characteristics of participants

**Figure 1:**
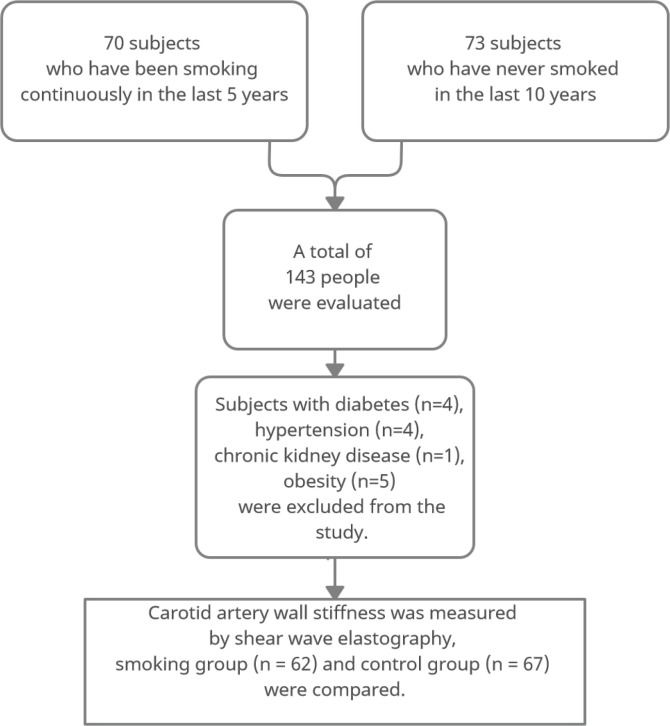
Flowchart of a cross-sectional study of male smokers and non-smokers screened, recruited, and analyzed for vascular wall stiffness, in a radiology clinic in Istanbul, 2020 (N=129)

In this cross-sectional study, vessel wall stiffness over the common carotid arteries of all participants was measured by the SWE method. In addition, blood pressure, fasting blood glucose, body mass index (BMI), HDL and LDL cholesterol measurements were recorded for the participants. The stiffness measurements of the distal half of the common carotid artery and the anterior wall of the patients who applied to our institution between September 2019 and September 2020 were evaluated using the SWE method. The study was carried out in the radiology clinic of a 400-bed training and research hospital. The subjects were selected from volunteers referred to the Radiology clinic for different reasons. All subjects were selected as male so that the effect of gender on the development of atherosclerosis did not affect the results. Sixty-two people who smoked at least five cigarettes a day continuously for at least five years were included in the study, and 67 people who had never smoked in the last ten years were included in the control group. First, smokers within a specific age range were taken, and measurements were made. Then, a control group in a similar age range was determined and measured. The mean age of the 62 smokers was 40.1 ± 5.4 years and the age range was 31–55 years; the mean age of the control group was 40.9 ± 5 years and the age range was 33–55 years. Participants with chronic diseases known to affect carotid intima-media thickness and arterial wall stiffness (diabetes mellitus, hyperlipidemia, chronic kidney disease, hypertension, heart disease, LDL/HDL ratio above 3.5, and body mass index, above the 95th percentile in age-appropriate curves) were excluded from the study (Figure 1). For this study, ethics committee approval was obtained in our institution in accordance with the Declaration of Helsinki.

### Shear wave elastography

In this elastography method based on acoustic radiation force impulse (ARFI), collimated and amplified ultrasound waves are sent into the tissue^22^. As a result of the pulse sent to the tissue with ARFI, shear waves are created in the target tissue that move horizontally perpendicular to this angle. Although there are different techniques, the two-dimensional SWE technique we used in our study was an imaging method obtained by simultaneously applying multiple ARFI waves into the tissue and measuring the resulting shear waves. The basis of imaging builds on the increase in the speed of the shear wave in that tissue as the stiffness of the tissue increases. This basic physics principle used in the elastography technique is based on a similar basis to the pulse wave velocity measurement used in the measurement of vessel wall stiffness in the past years. With the shear wave velocity measurement method, the measurement units used in elastography can be obtained as Young’s modulus (kPa) and shear wave velocity (m/s). The measurement is executed in the form of velocity measurement (m/s) and conversion between units. This conversion is performed mathematically by an equation in a program^22^. In our study, the kilopascal (kPa) was used as the unit of tissue stiffness. The interquartile range (IQR)^23^ calculates the variability between measurements. The ratio of IQR to median value of valid measurements (IQR/median) is aimed to be <30%. At least four measurements are required to see the IQR/median value on the Philips device that we use. For this reason, 4–8 measurements were made in each subject. If the IQR/median ratio of the measurements was <30%, they were considered valid and averaged.

### Study protocol

**Figure 2:**
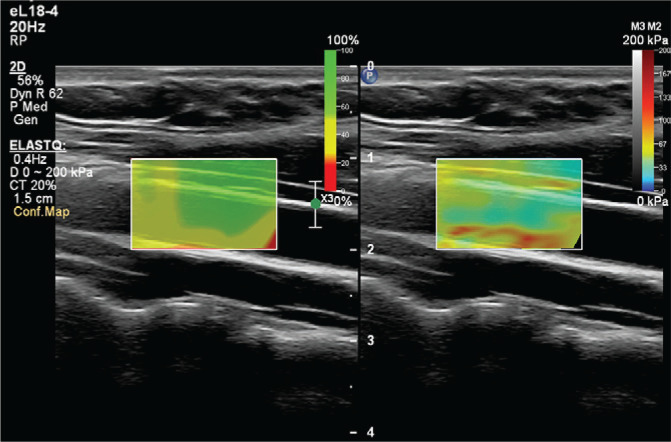
Confidence map indicates safe areas (in green) for measurement

**Figure 3:**
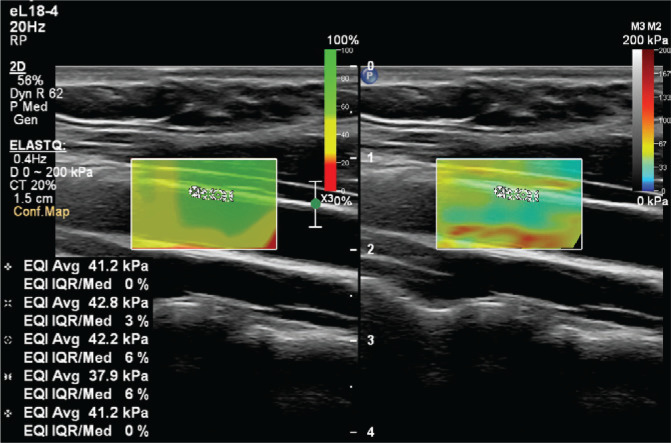
In SWE measurements made on the intima-media complex on the anterior wall of the common carotid artery, the aim should be to obtain a low IQR/median ratio, which indicates the reliability of the measurement

To prevent the acute effects of cigarettes and dietary nitrates from affecting the measurements, the subjects stopped consuming food, cigarettes, alcohol, and caffeine-containing beverages for at least 8 hours before the examination. After the subjects rested for at least 5 minutes, the examination was conducted in a quiet room. An experienced radiologist performed the examinations. Philips EPIQ Elite (Philips Healthcare, Andover, MA, US) device was used in all examinations. All subjects were placed in a supine position with their head in a neutral position. Measurements were preferably made in the distal 1/3 of the right common carotid artery, from the anterior wall, with a 7.5 MHz linear probe (Figures 2 and 3). The ROI (region of interest) for measurement was sized and positioned to be as centralized as possible without extending beyond the intima-media complex of the carotid artery wall. Brachial blood pressure (BP) was recorded in the supine position using an automated digital oscillometric BP monitor (Omron 705-CP-E; Omron Corp., Tokyo, Japan) in a quiet room at room temperature after the subject rested for 5 minutes. Two measurements, separated by 1-minute intervals, were made, and the average was recorded. Data were obtained for the participants’ fasting blood sugar, body mass index (BMI), HDL and LDL cholesterol values, using the records on the hospital information system.

### Statistical analysis

The IBM SPSS Statistics 22 (IBM Corp., Armonk, NY, USA) program was used for statistical analysis. Conformity of the parameters to the normal distribution was evaluated with the Shapiro-Wilks test, and the parameters were found to follow a normal distribution. For the quantitative data, descriptive statistical methods (mean, standard deviation, frequency) were used. Student’s t-test was used to compare parameters between two groups in comparing quantitative data, and continuity (Yates) correction was used to compare qualitative data. Significance was evaluated at the p<0.05 level.

## RESULTS

Male subjects aged 30–55 years were included in the study. The age range of 62 smokers included in the study was 31–55 years, and their mean age was 40.1 ± 5.4 years. The age range of 67 subjects in the control group was 33–55 years, and their mean age was 40.9 ± 5.0 years. The mean of SWE measurements made over the intima-media complex of the common carotid arteries of the smokers was 47.3 ± 6.2 kPa, and of the control group was 42.9 ± 4.0 kPa. The mean diastolic blood pressure of the smokers was 82.5 ± 6.5 mmHg, and of the control group was 81.7 ± 5.0 mmHg. The mean fasting blood sugar of smokers was 99 ± 7.7 mg/dL, and of the control group was 98.5 ± 7.2 mg/dL. The mean body mass index (kg/m^2^) of the smokers was 25.9 ± 2.6, and of the control group was 26.2 ± 2.9. The mean HDL of the smokers was 46.9 ± 5.6 mg/dL, and of the control group was 50.3 ± 5.1 mg/dL. The mean LDL of the smokers was found to be 147.3 ± 9.3 mg/dL, and of the control group was 136.9 ± 5.9 mg/dL (Table 1).

**Table 1:** Basic characteristics and carotid artery shear wave elastography values of male smokers and control group in a radiology clinic in Istanbul, 2020 (N=129)

*Characteristics*	*Smokers (N=62) mean ± SD*	*Control group (N=67) mean ± SD*	*p*
Age (years)	40.1 ± 5.4	40.9 ± 5.0	0.352
Diastolic blood pressure (mmHg)	82.5 ± 6.5	81.7 ± 5.0	0.444
Fasting blood glucose (mg/dL)	99.0 ± 7.7	98.5 ± 7.2	0.690
Body mass index (kg/m^2^)	25.9 ± 2.6	26.2 ± 2.9	0.483
HDL cholesterol (mg/dL)	46.9 ± 5.6	50.3 ± 5.1	0.02
LDL cholesterol (mg/dL)	147.3 ± 9.3	136.9 ± 5.9	0.01
CCA stiffness (kPa)	47.3 ± 6.2	42.9 ± 4.0	0.01

Student’s t-test.

* p<0.05.

Carotid artery wall stiffness measured by SWE was statistically significantly higher in smokers (47.3 ± 6.2 kPa) compared to the control group (42.9 ± 4 kPa) (p<0.05). Similarly, LDL cholesterol values were statistically significantly higher in smokers compared to the control group, while HDL cholesterol values were statistically significantly lower in smokers (p<0.05). Parameters including age, diastolic blood pressure, body mass index, and blood glucose values did not show a statistically significant difference between smokers and non-smokers (Table 1).

## DISCUSSION

We used shear wave ultrasonography to measure the carotid artery wall stiffness in smokers and in the control group, to investigate the impact of chronic smoking. Smokers’ arterial wall stiffness values were statistically considerably higher than those of non-smokers.

The pathological process responsible for the development of cardiovascular diseases is mainly associated with endothelial dysfunction^24^. It is thought that smoking reduces basal NO production and thus causes endothelial dysfunction. NO is a potent smooth muscle relaxant and principal regulator of arterial smooth muscle tone^25^. Histological changes occur before clinical signs, and if the necessary precautions are not taken, diseases occur later in life. To prevent morbidity and mortality caused by cardiovascular diseases, it has become important in recent years to determine the changes that are not reflected in the clinic, through carotid intima-media thickness and arterial wall stiffness. The progression of the disease can be reduced by detecting endothelial dysfunction and atherosclerosis that may occur with smoking in the early period, by measuring carotid intima-media thickness and arterial wall stiffness^26,^ ^27^.

Mahmud and Feely^16^ demonstrated that smoking just one cigarette can cause an abrupt rise in arterial stiffness, and found that smokers had significantly higher levels of Aix. According to Barua et al.^11^, light and heavy smokers had similar impairments in basal NO production as well as eNOS expression and activity. There is some evidence to suggest that quitting smoking improves endothelial function. According to one study, there was no difference in plasma NO levels between never smokers and ex-smokers, but there was a significant reduction among active smokers^28^. No relationship was found between basal plasma NO levels and the duration of time, varying from six months to twelve years, that elapsed since ex-smokers had quit. This implies that the first few months following quitting smoking may see an improvement in endothelial function^28^.

The relationship between the development of atherosclerosis and increased arterial wall stiffness has been demonstrated by many studies^29,30^. An increase in arterial wall stiffness has been reported in various conditions such as hypertension, stroke, and heart failure^29-31^. In the past years, many studies have reported increased arterial wall stiffness in smokers. Numerous studies have been conducted to reveal early endothelial dysfunction using different methods, such as the pulse wave method (PWV), flow-mediated dilation (FMD), and ankle-brachial pressure index (ABPI) for vessel wall stiffness analysis. Thus, decreased FMD and increased PWV values reflecting increased arterial wall stiffness were demonstrated in smokers^32,33^. In our study, we used the elastography method, which is increasingly used in evaluating arterial wall stiffness in smokers and is thought to allow reliable, rapid, and repeatable measurements. We found an increase in carotid artery wall stiffness in smokers compared to healthy controls.

Increased intima-media complex thickness is an accepted marker for early atherosclerosis and endothelial dysfunction^34^. Many authors investigated intima-media complex thickness in smokers and reported increased intima-media complex thickness^35^. The increase in thickness of the intima-media complex formed in the arterial wall is expected to increase the arterial wall stiffness.

In contrast to techniques such as FMD, ABPI, and PWV, which measure peripheral elastic modulus in arterial wall stiffness measurement, SWE evaluates the elastic modulus of the vessel wall^36^. Thus, SWE allows time-related, motion-based measurement from multiple points in assessing the arterial wall stiffness of the segment being examined. Consequently, it provides a precise topographic definition, including space and time dimensions, thus providing superior information about arterial wall stiffness^36^.

### Limitations

In this study, LDL cholesterol levels were higher, and HDL cholesterol levels were lower in smokers compared to the control group. The effects of chronic smoking on blood lipids have been known for a long time. However, due to the nature of our study, the relationship between abnormal blood lipid levels and smoking was not evaluated. For this reason, it has not been fully revealed to what extent the abnormality in blood lipids, together with or independently of smoking, is the cause of vessel wall stiffness. This is one of the important limitations of our study. There were other limitations in our study. First, there were relatively few participants in our study. Secondly, although we included smokers in the study within a certain rule in terms of time and number of cigarettes, we did not rank smoking according to time and number of cigarettes; therefore, we did not evaluate the correlation between smokers’ arterial wall stiffness and the amount and time of smoking. In addition, we did not compare SWE with other methods used to measure arterial wall stiffness, such as FMD and PWV. Although many studies show that tissue stiffness measurements with SWE are reliable and reproducible, a comparative study of SWE, PWV, and FMD should be done to measure arterial wall stiffness in smokers. In addition, although we tried to exclude diseases that may affect the intima-media complex thickness and wall stiffness of the carotid artery, as far as possible, it was not possible to identify and exclude all conditions associated with vessel wall stiffness. We can also add a non-causal design and inclusion of only male subjects. Finally, we evaluated only the stiffness of the carotid artery. For this reason, conducting studies measuring the stiffness of different arteries in smokers and comparing the results will provide valuable information.

## CONCLUSIONS

Chronic smoking is found to increase arterial wall stiffness. Although there are studies on the determination of subclinical atherosclerosis in smokers, there is no study including the SWE method. In this study, it was shown with the SWE method that smoking, which is one of the most critical risk factors for cardiovascular diseases, can cause arterial stiffness and, therefore, subclinical atherosclerosis. Thus, by examining larger groups, subclinical atherosclerosis can be detected early.

## Data Availability

The data supporting this research are available from the authors on reasonable request.
